# The complete chloroplast genome sequence of horticultural plant, *Impatiens hawkeri* (Sect. Balsaminacea, Impatiens)

**DOI:** 10.1080/23802359.2019.1698339

**Published:** 2019-12-11

**Authors:** Chao Luo, Wulue Huang, Yang Li, Zhixi Feng, Jiapeng Zhu, Yingli Liu, Zhenkai Tong, Yan Liang, Haiquan Huang, Meijuan Huang

**Affiliations:** a College of Landscape Architecture and Horticultural Science, Southwest Forestry University, Kunming, China;; b Research and Development Center of Landscape Plants and Horticulture Flowers, Southwest Forestry University, Kunming, China

**Keywords:** *Impatiens hawkeri*, chloroplast, genome sequence, horticultural plant

## Abstract

The complete chloroplast genome sequence of *Impatiens hawker*, a widely cultivated horticultural species in the world is 151,692 bp, with a typical quadripartite structure including a pair of inverted repeat (IRs, 25,584 bp) regions separated by a small single copy (SSC, 17,494 bp) region and a large single copy (LSC, 83,029 bp) region. The overall GC content of *I. hawker* plastid genome was 36.8%. The whole chloroplast genome contains 135 genes, including 89 protein-coding genes (PCGs), 38 transfer RNA genes(tRNAs), and 8 ribosomal RNA genes (rRNAs). Among these genes, 15 genes have one intron and 2 genes contain two introns. To investigate its evolution status, the phylogenetic tree based on APGIII reveal that there are close relationships to the same genus species *I. uliginosa* and *I. piufanensis*.

The largest angiosperm genus impatiens including the approximately 1,000 known species, most live in the wild and commonly as understory woodland plants with 250 species in China (Grey-Wilson 1980; Yu et al. 2016). *Impatiens* stems and roots have been isolated and identified in traditional chinese medicine (TCM) for treatment of a wider range of diseases and aiments, including lumbago, neuralgia, burns and scalds (Janssens et al. 2012; Mohammad et al. 2012; Li et al. 2015). *I. hawkeri* is an annual shade-tolerant flower used extensively in display gardens, landscape beds, container gardens and hanging baskets (Salgado et al. 2018). Though there are many pharmacology studies done on the plant, not much of molecular genetic informatics is currently available (Rahelivololona et al. 2018). For a better understanding of *I. hawkeri,* it is essential to reconstruct a phylogenetic tree of the *Impatiens* species based on high-throughput sequencing approaches (Luo et al. 2019).

The fresh leaves of *I. hawkeri* were obtained from the nursery of Southwest Forestry University (Yunnan, China; Coordinates: 102°76′43″E, 25°06′15″N; Altitude: 1953.7 m). Total genomic DNA was extracted with the Omega Plant Genomic DNA Preps Kit. The certificate specimens of *I. hawkeri* samples were properly deposited at the herbarium of Southwest Forestry University (NO. SWFU-IBXJNY20180811) and DNA samples were properly stored in College of Landscape Architecture and Horticultural Science, Southwest Forestry University, Kunming, Yunnan, China. Total genomic DNA was used to generate libraries with an average insert size of 400  bp and sequences using the Illumina Hiseq X platform. Approximately 7.68 GB of raw data were generated with 150 bp paired-end read lengths. the complete chloroplast genome used the software of the program Geneious R10 by the raw data with *I. pinfanensis* (GenBank_MG162586.1) as the reference (Jin et al. 2018). Genome annotation was performed with the DOGMA (Wyman et al. 2004). The cpDNA sequence with complete annation information was deposited at GenBank database under the accession number MN687854.

The plastome of *I. hawkeri* is 151,692bp, a double stranded circular DNA with a pair of inverted repeats (IRA and IRB) of 25,584bp, a large single copy (LSC) region of 83,029bp and a small single copy (SSC) region of 17,494bp. The junction between IRB and SSC is 50 bp within the *ndhF* gene. The junction between SSC and IRA is within the *ycf1* gene, which is 5,508bp long, of which 4327 bp lies in the SSC. The overall GC content of the whole plastome was 36.8%, while the corresponding values of the LSC, SSC, and IR regions were 34.5%, 29.6%, and 43.2%, respectively. The complete chloroplast plastome annotated 136 genes, including 89 protein-coding genes (PCGs), 38 tRNA genes and 8 rRNA genes. Among these genes, 15 genes have 1 intron and 2 genes contain 2 introns.

To analyze the phylogenetic location of *I. hawkeri* .13 chloroplast genome sequences of Ericales were aligned by the MAFFT version 7 software (Katoh and Standley 2013). A maximum likelihood method for phylogenetic analysis was made base on GTR + I + G model in the RAxML version 8 program with 1000 bootstrap replicates (Darriba et al. 2012; Stamatakis 2014). The phylogenetic tree was divided into two parts, which is in agreement with previously studies (Li et al. 2018), and reveal that there are close relationships to the same genus species *I.uliginosa* (MN533984), *I. piufanensis* (MG162586.1) and *Hydrocera triflora* (MG162585.1) (Figure 1).

**Figure 1.**
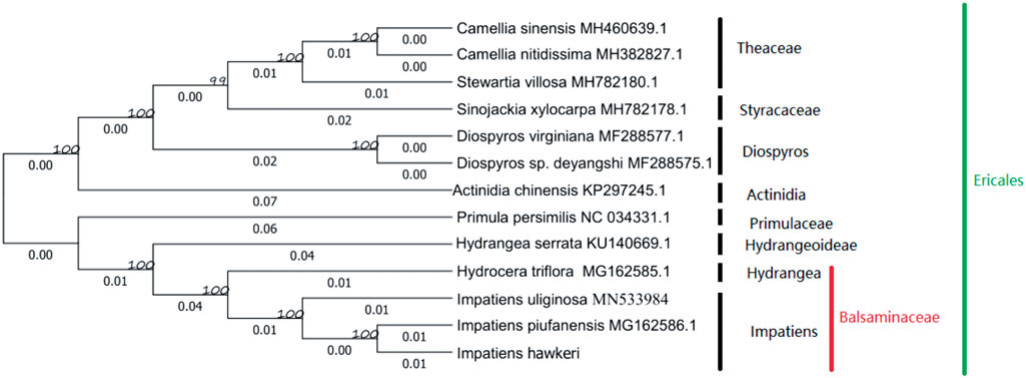
The ML phylogenetic tree for *I. hawkeri* based on 12 chloroplast genome sequences of Ericales. Numbers on the nodes are bootstrap values from 1000 replicates. Accession numbers: *Camellia sinensis* (MH460639.1), *Camellia nitidissima* (MH382827.1), *Stewartia villosa* (MH782180.1), *Diospyros virginiana* (MF288577.1), *Diospyros sp.deyangshi* (MF288575.1), *Sinojackia xylocarpa* (MH782178.1), *Actinidia chinensis* (KP297245), *Primula persimilis*(NC034331.1), *Hydrangea serrata*(KU140669.1), *Hydrocera triflora* (MG162585.1), *Impatiens uliginosa* (MN533984) and *Impatiens piufanensis* (MG162586.1).
